# C2-Ceramide Induces Cell Death and Protective Autophagy in Head and Neck Squamous Cell Carcinoma Cells

**DOI:** 10.3390/ijms15023336

**Published:** 2014-02-21

**Authors:** Wenyuan Zhu, Xinhua Wang, Yi Zhou, Huiming Wang

**Affiliations:** 1 Department of Oral and Maxillofacial Surgery, the First Affiliated Hospital, Zhejiang University, Hangzhou 310003, China; E-Mail: zwy555star@163.com; 2 Department of Oral and Maxillofacial Surgery, the Affiliated Hospital of Stomatology, College of Medicine, Zhejiang University, Hangzhou 310003, China; 3 Department of Oral Implantology, the Affiliated Hospital of Stomatology, College of Medicine, Zhejiang University, Hangzhou 310003, China; E-Mails: wxhalex@163.com (X.-H.W.); zhouyizyzyzy@163.com (Y.Z.)

**Keywords:** head and neck squamous cell carcinoma, C2-ceramide, apoptosis, necroptosis, autophagy, ERK1/2 pathway

## Abstract

Ceramides are second messengers involved in several intracellular processes in cancer cells, amongst others. The aim of this study was to evaluate the anti-tumor efficacy of C2-ceramide (C2-Cer; *N*-acetyl-d-sphingosine) by investigating cell death and autophagy in head and neck squamous cell carcinoma (HNSCC) cells. C2-Cer showed concentration-dependent cytotoxicity in HN4 and HN30 cell lines. It simultaneously induced caspase-3-independent apoptosis and programmed necrosis. C2-Cer markedly increased the expression level of microtubule-associated protein 1 light chain 3B (LC3B) type II associated with protective autophagy. An autophagy inhibitor enhanced C2-Cer-mediated cytotoxicity, while a programmed-necrosis inhibitor produced the opposite effect. Furthermore, C2-Cer up-regulated the phosphorylation of extracellular signal-regulated kinase 1/2, but down-regulated its downstream substrate phospho-mammalian target of rapamycin (p-mTOR) during the autophagy process. These results suggested that C2-Cer exerts anti-tumor effects by inducing programmed apoptosis and necrosis in HNSCC, and these cytotoxic effects are enhanced by an autophagy inhibitor.

## 1. Introduction

Head and neck squamous cell carcinoma (HNSCC) accounts for 90% of head and neck cancers, and is the fifth most common cancer diagnosed worldwide. It originates in cells that form the lining of the mouth, nose, throat, ear, or the surface layer covering the tongue [1]. HNSCC is responsible for 500,000 cancer-related deaths annually worldwide [2]. The overall incidence of cancer has declined in the United States, Canada, and Western Europe within the past 20 years as a result of reduced tobacco and alcohol consumption [3–7]. Furthermore, the human papillomavirus (HPV) has recently received considerable attention as a causative factor of oropharyngeal cancer [8]. Treatment for HNSCC consists mainly of a combination of surgery and radiation, or combined radiation and chemotherapy. Platinum-based agents provide the backbone of the standard chemotherapeutic regimens for HNSCC. These agents include cisplatin, which is widely-used in chemotherapy, as well as carboplatin and oxaliplatin [9]. Although treatment strategies have improved significantly in recent decades, the 5-year survival rate remains about 50%. Therapeutic management of local, locally-advanced, recurrent, and metastatic HNSCC is often limited by resistance to chemotherapy and radiotherapy, as well as by unacceptable toxicity and side-effect profiles [10]. Some evidence has indicated that autophagy promotes cancer cell resistance to chemotherapy and radiotherapy, and the abrogation of autophagy through autophagy inhibitors or knockdown of autophagy-related molecules potentiates the resensitization of therapy-resistant cancer cells to anti-cancer treatment [9].

Autophagy is a major intracellular pathway for the degradation and recycling of long-lived proteins, lipid droplets, protein aggregates, mature ribosomes, glycogen, and even entire organelles, such as the endoplasmic reticulum, mitochondria, and Golgi apparatus, thereby maintaining homeostasis and viability during periods of metabolic stress [11]. As an essential cellular process, autophagy has been shown to be involved in cancer development and progression in a variety of ways. Interestingly, it has been considered to be a “double-edged sword” in anti-cancer therapy. Autophagy enhances the degradation of proteins and organelles to provide amino acids, fatty acids, and nucleotides for reuse, leading to cancer cell survival under unfavorable metabolic conditions, such as starvation, hypoxia, and radiation [12]. Promising targeted agents including cetuximab, induce stresses to activate prosurvival-autophagy [13]. Paradoxically, unrestrained autophagy may lead to cell death by cellular self-degradation, and autophagy itself can induce caspase-3-independent programmed cell death in cancer [14,15].

Sphingolipids have emerged as bioeffector molecules, controlling various aspects of cell growth and proliferation in cancer. These lipid molecules have also been implicated in the mechanism of action of cancer chemotherapeutics. As the central molecule of sphingolipid metabolism, ceramides, are sphingolipid second messengers involved in a wide variety of cell processes [16,17]. Exogenous ceramides have the ability to mimic these functions in cells. As pro-apoptotic mediators, ceramides have been widely implicated in neurodegenerative diseases, such as Alzheimer’s disease. Ceramides can trigger autophagy in the presence of extracellular nutrients. In cancer research, ceramides have been shown to induce apoptosis and autophagy, which has been a focus of cancer therapy in terms of carcinogenesis, proliferation, and metastasis in diseases such as colon carcinoma, hepatocarcinoma, and leukemia [18–22]. Ceramide metabolism has shown clinical relevance in the pathogenesis of HNSCC, and chemotherapeutic drugs, such as gemcitabine and doxorubicin, have been reported to elevate cellular ceramide levels [23–25]. However, whether ceramides contribute to the pro-survival response or to the anti-tumor effect of chemotherapy in HNSCC cells is unknown. This study investigated the effects of the C2-ceramide (C2-Cer; *N*-acetyl-d-sphingosine) on HNSCC cell proliferation and the possible mechanisms responsible for these effects.

## 2. Results and Discussion

### 2.1. Results

#### 2.1.1. C2-Cer Induced Apoptosis in HNSCC

**Figure 1.**
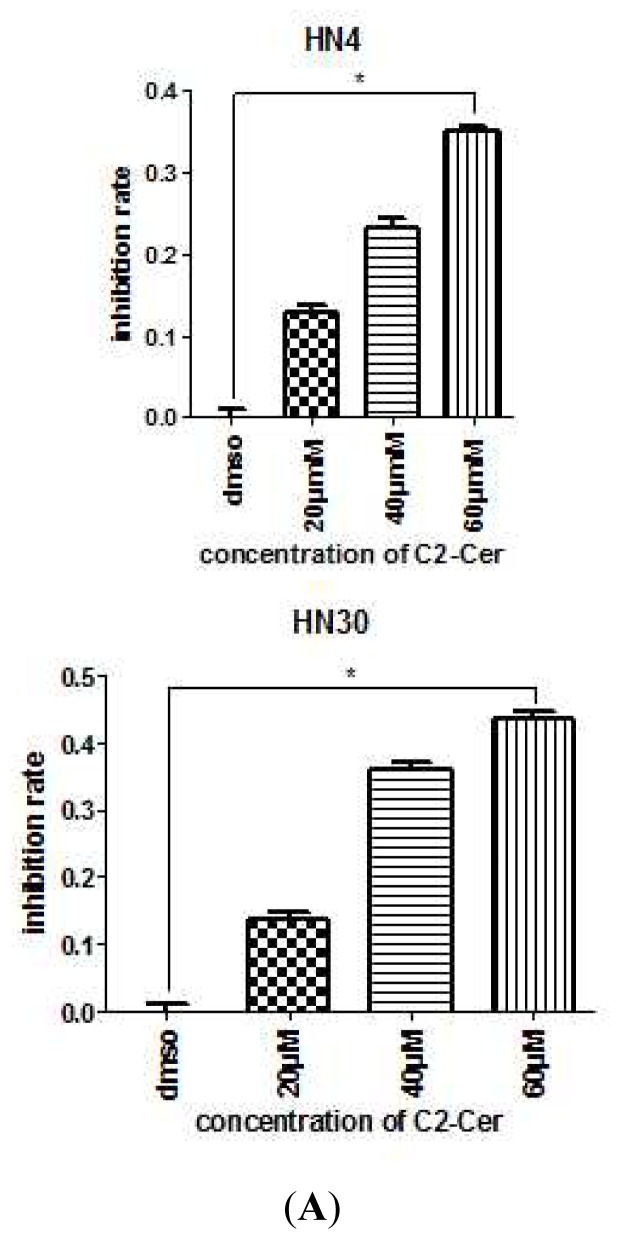

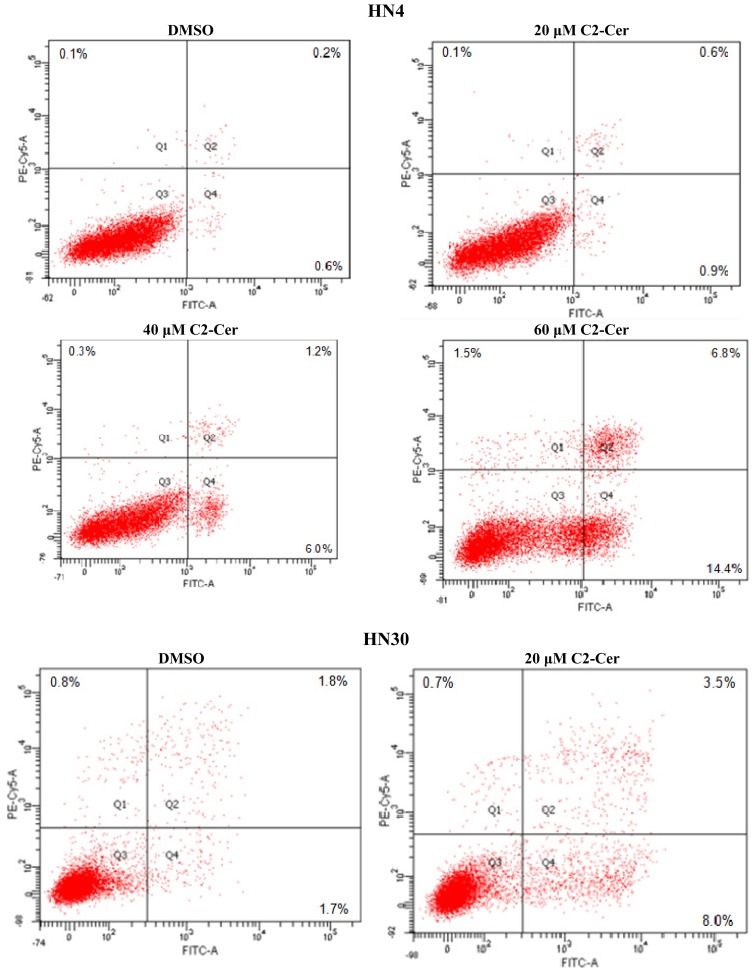

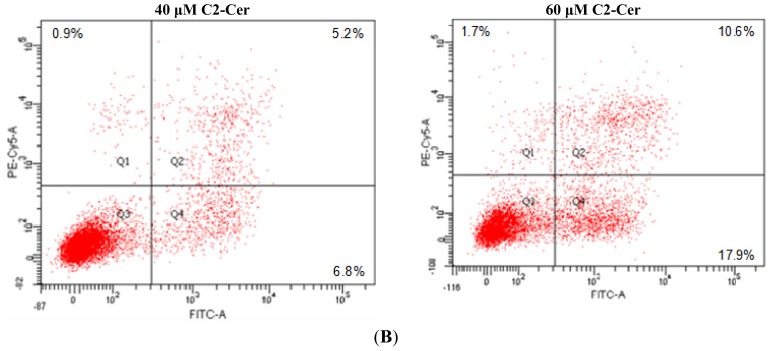
(**A**) Cell Counting Kit-8 (CCK8) assay. Cells were treated with different concentrations (0, 20, 40, 60 μM, respectively) of C2-Cer for 24 h. The cell proliferation inhibition rate increased after treatment with C2-Cer in a concentration-dependent manner; (**B**) Flow cytometry. Cells were treated with different concentrations (0, 20, 40, 60 μM, respectively) of C2-Cer for 24 h. Q2 + Q4 represent apoptotic cells. Apoptotic cells were significantly increased when exposed to C2-Cer in a concentration-dependent manner. (^*^: *p* < 0.05).

**Figure 2.**
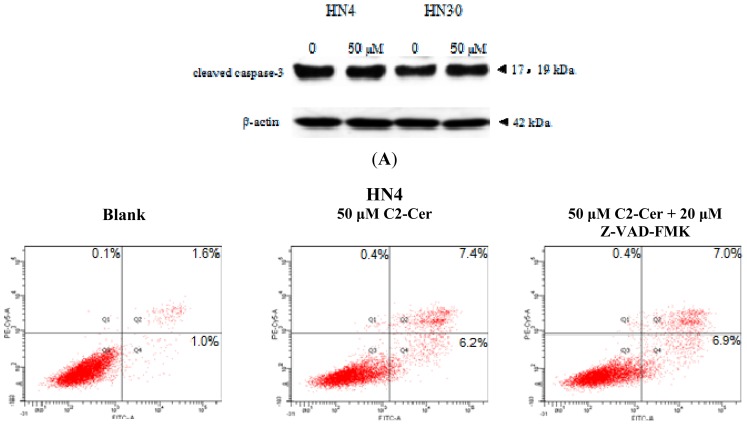

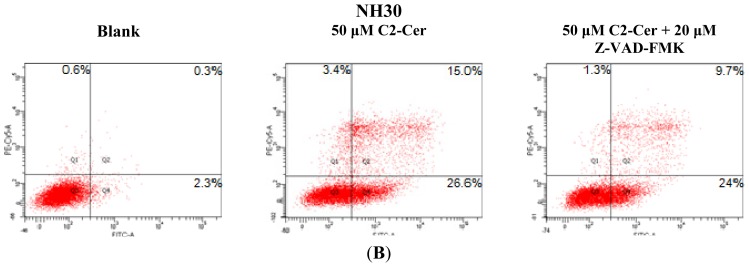
(**A**) Western blotting. Expression levels of caspase-3 were unaffected by treatment with C2-Cer (0 and 50 μM, respectively); (**B**) Flow cytometry. Cells were treated with 0, 50 μM C2-Cer and 50 μM C2-Cer plus 20 μM Z-VAD-FMK for 24 h. No difference in apoptotic cell numbers was established between cell treated with C2-Cer treated only and co-treated cells.

HN4 and HN30 cells were treated with various concentrations of C2-Cer for 24 h, and cell viability was assessed to determine the cytotoxic effects of C2-Cer. All cells showed morphological changes, including cell shrinkage, detachment from other cells, and formation of vesicles in the cytoplasm within 24 h of treatment, as observed via light microscopy. More C2-Cer-treated cells floated in the treated medium compared with control cells. C2-Cer significantly inhibited the proliferation of HNSCC cells in a concentration-dependent manner (Figure 1A; *p <* 0.05). The cytotoxic effect declined as the concentration increased. The LD_50_ in HN30 cells was 60 μM, though HN4 cells were less sensitive to C2-Cer than HN30 cells. Flow cytometric analysis showed that the ratios of propidium iodide (PI)+ cells were significantly higher in C2-Cer-treated cells than in controls in a concentration-dependent manner. C2-Cer induced apoptosis in both cell lines (Figure 1B). The expression level of cleaved caspase-3 was measured by Western blotting to determine the apoptosis mechanism of C2-Cer. There was no significant change in the level of caspase-3 cleavage after C2-Cer treatment (Figure 2A). To verify that apoptosis was induced by C2-Cer via a caspase-3-independent pathway, the pan-caspase inhibitor Z-VAD-FMK was added to C2-Cer-treated cells. Flow cytometric analysis showed that apoptosis was not inhibited by Z-VAD-FMK (Figure 2B).

#### 2.1.2. C2-Cer Induced DNA Fragmentation of HNSCC

**Figure 3.**
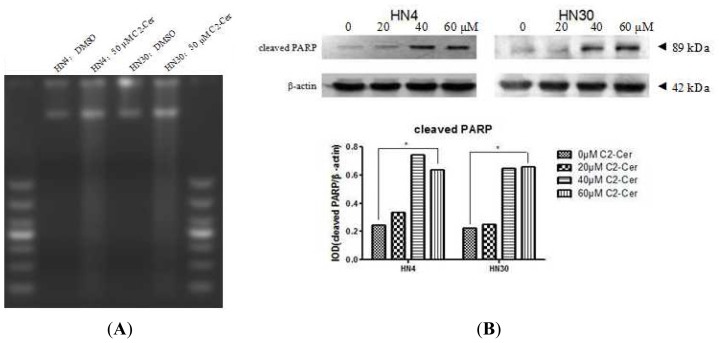

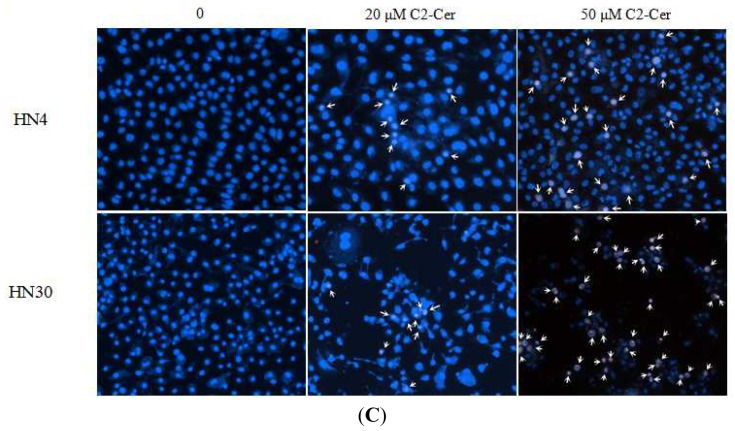
(**A**) DNA fragmentation assay. Cells were treated with dimethyl sulfoxide (DMSO) and 50 μM C2-Cer. DNA was extracted and subjected to 2 g/L agarose gel electrophoresis. DNA from C2-Cer-treated cells showed fuzzy and continuous DNA ladders; (**B**) Western blotting. Cleaved poly (ADP-ribose) polymerase (PARP) was increased after treatment with C2-Cer (0, 20, 40, 60 μM, respectively) compared with controls; (**C**) Hoechst 33342/PI double staining. Cells were treated with different concentrations of C2-Cer (0, 20, 50 μM, respectively). Necrotic cells were observed after exposure to C2-Cer in a concentration-dependent manner (necrotic cells, Hoechst 33342+/PI+, arrow). (^*^: *p* < 0.05).

DNA strand breaks were detected by DNA agarose gel electrophoresis. DNA from control cells treated with dimethylsulfoxide (DMSO) maintained its integrity, with no DNA ladder formation. However, cells treated with C2-Cer developed fuzzy and continuous DNA laddering, indicating necrotic DNA fragmentation (Figure 3A). The poly (ADP-ribose) polymerase (PARP) protein family is involved in a number of cellular processes involving DNA repair and programmed cell death. Cleaved PARP protein levels were increased by C2-Cer, indicating activation of PARP-associated DNA repair (Figure 3B).

#### 2.1.3. C2-Cer Induced Programmed Necrosis in HNSCC Cells

**Figure 4.**
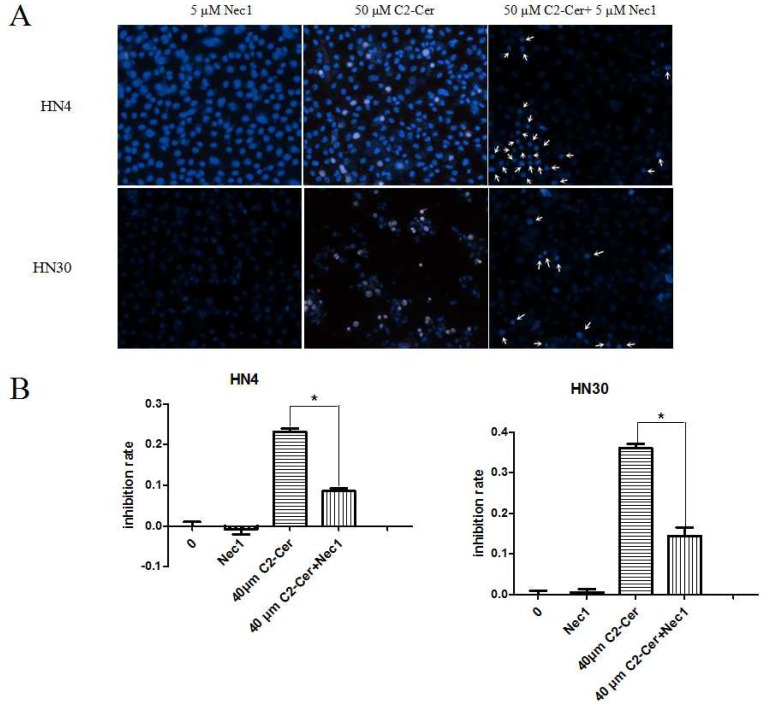
(**A**) Cells were treated with 5 μM necrostatin-1 (Nec-1), 50 μM C2-Cer, or 40 μM C2-Cer plus 5 μM Nec-1 for 24 h, stained with Hoechst 33342 and PI. No necrotic cells were observed after treatment with Nec-1 except apoptotic cells (apoptotic cells, Hoechst 33342−/PI++, arrow). The inhibition rate of cell proliferation declined after Nec-1 treatment (*p <* 0.05); (**B**) Cells were treated with 5 μM Nec-1, 40 μM C2-Cer, or 40 μM C2-Cer plus 5 μM Nec-1 for 24 h. The viability of the co-treated cells was higher than that of the C2-Cer treated cells as assessed via the CCK8 assay. (^*^: *p* < 0.05).

Following agarose gel electrophoresis, DNA was visualized by Hoechst 33342/PI immunofluorescence double staining to determine the induction of programmed necrosis by C2-Cer. C2-Cer increased the incidence of Hoechst 33342+/PI++ cells in a concentration-dependent manner, indicating the induction of programmed necrosis. Double positive cells were markedly decreased by co-treatment with the necroptosis inhibitor necrostatin-1 (Nec-1), leaving only PI++ cells, indicative of apoptosis (Figures 3C and 4A). The viability of cells exposed to C2-Cer was increased after Nec-1 treatment, as assessed with the Cell Counting Kit-8 (CCK8) assay. These results indicated that Nec-1 blocked programmed necrosis and significantly weakened C2-Cer-mediated cytotoxicity (Figure 4B; *p* < 0.05).

#### 2.1.4. C2-Cer Induced Autophagy

**Figure 5.**
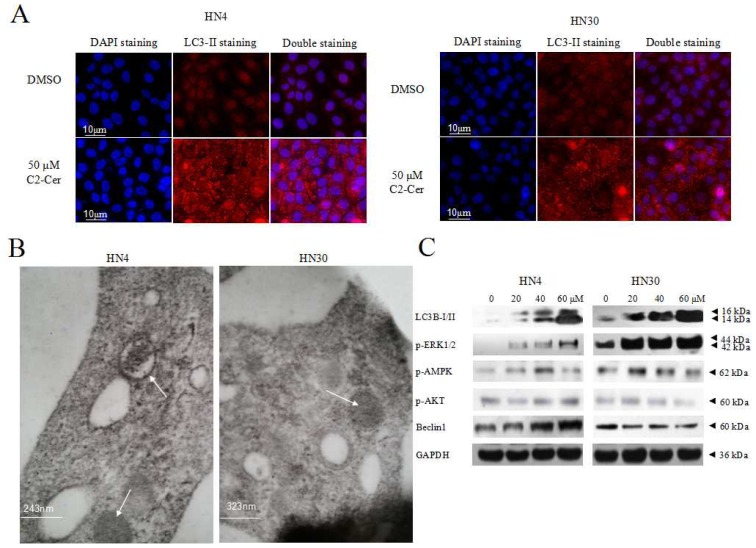
(**A**) DAPI/LC3-II (4′,6-diamidino-2-phenylindole/microtubule-associated protein 1 light chain 3-II) double staining. LC3-II-stained particles accumulated in the cytoplasm of treated cells compared with control cells; (**B**) Electron microscopy. Cells were treated with 50 μM C2-Cer for 4 h. Specific autophagic vacuoles accumulated in the cytoplasm (×30,000, arrow); (**C**) Western blotting. Expression levels of autophagy-associated proteins (LC3B-II, phospho-extracelluar signal-regulated kinase 1/2 (p-ERK1/2), phospho-AMP-activated protein kinase (p-AMPK), p-Akt, Beclin 1) were analyzed. Levels of LC3B-II and p-ERK1/2 were elevated after treatment of cells with C2-Cer. Other protein levels remained unaffected by C2-Cer treatment.

**Figure 6.**
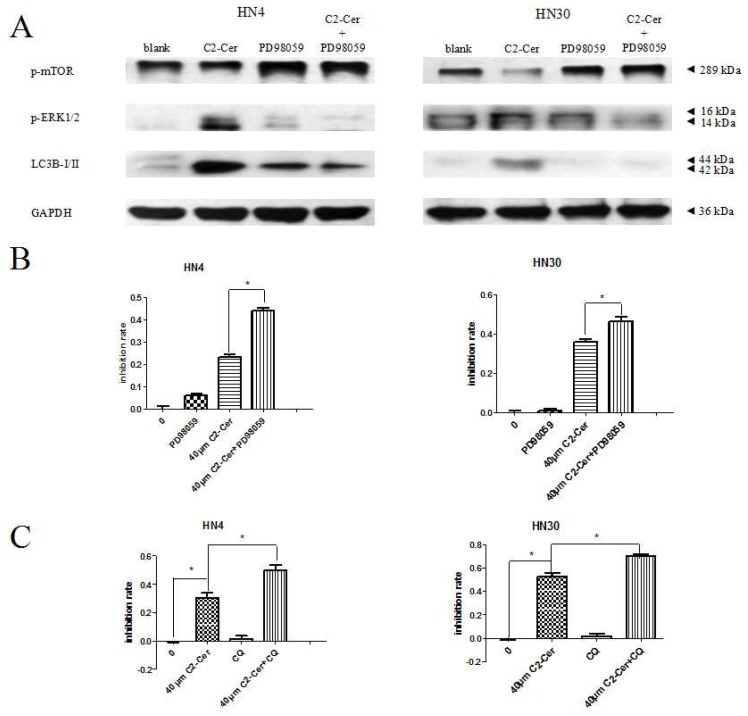
(**A**) Western blotting. Expression levels of LC3B-II, phospho-mammalian target of rapamycin (p-mTOR) and phospho-extracelluar signal-regulated kinase 1/2 (p-ERK1/2) were analyzed after treatment with PD98059/C2-Cer. LC3B-II and p-ERK1/2 levels declined and p-mTOR levels were elevated compared with cells treated with C2-Cer only; (**B**) CCK8 assay. Treatment with 10 μM PD98059 significantly reduced the viability of cells exposed to C2-Cer; (**C**) CCK8 assay. CQ enhanced the growth-inhibition effect of C2-Cer compared with C2-Cer only. (^*^: *p* < 0.05).

Microtubule-associated protein 1 light chain 3 (LC3) -II transforms from LC3-I and acts as a marker for autophagic vesicles and autophagic activity, and LC3B-II (isoform of LC3-II) is correlated with elevated levels of autophagic vesicles. Endogenous LC3-II levels increased in HNSCC cells within 24 h after C2-Cer treatment in a concentration-dependent manner. LC3-II levels were higher in cells co-treated with the autophagy inhibitor chloroquine (CQ) compared with cells treated with C2-Cer alone. This result was confirmed by immunofluorescence. Fluoresence microscopic evaluation of LC3-II antibody-prestained cells revealed more stained particles in the cytoplasm of treated cells compared with control cells (Figure 5A). Specific autophagic vacuoles were observed in the cytoplasm via electron microscopy, 4 h after C2-Cer treatment (Figure 5B). LC3-II and phospho-extracelluar signal-regulated kinase 1/2 (p-ERK1/2) levels increased in concentration-dependently. However, the levels of phospho-AMP-activated protein kinase (p-AMPK), p-Akt and Beclin 1 showed no apparent differences, suggesting that autophagy may be induced through an ERK-related pathway (Figure 5C). Co-treatment with the mitogen-activated protein kinase kinase (MEK) inhibitor PD98059 reduced the expression levels of LC3B-II and p-ERK1/2, but increased p-mTOR (Figure 6A). In addition, cell viability was significantly reduced by PD98059, as shown via the CCK8 assay (Figure 6B; *p <* 0.05). These results suggest that PD98059 inhibited autophagy and increased the sensitivity of HNSCC cells to C2-Cer exposure.

#### 2.1.5. Autophagy Inhibition Sensitized Cells to C2-Cer-Mediated Cytotoxicity

CQ is a late-phase autophagy inhibitor that prevents autophagosomal degradation. In a CCK-8 assay, cells treated with CQ only showed no growth inhibition, indicating that CQ had no significant effect on the proliferation of HNSCC cells. However, CQ together with C2-Cer significantly reduced the viability of HNSCC cells compared with cells treated with C2-Cer only (Figure 6C; *p* < 0.05). These results suggest that the autophagy inhibitor CQ could sensitize HNSCC cells to C2-Cer-induced cytotoxicity.

### 2.2. Discussion

Ceramides are intracellular second messenger molecules that relay and amplify signals from cell surface receptors, resulting in potentially large changes in biochemical activities within cells. Emerging evidence suggests that ceramides with different fatty acid chain lengths might have distinct functions in the regulation of tumor growth and responsiveness to therapy. Reduced ceramide levels have been related to cell motility in premalignant oral lesions and some anticancer drugs, such asdeguelin, have been shown to increase cellular ceramide levels through *de novo* synthesis to mediate HNSCC cell death and apoptosis [25,26]. C2-Cer is a non-physiological short-chain ceramide that is used as a cell-permeable ceramide analog. The inhibitory effects of C2-Cer on cell proliferation and apoptosis have been shown in many leukemia and hepatocarcinoma cell types [20,21,27–29]. The results of the current study showed that growth and viability of HN4 and HN30 cells were inhibited dramatically by treatment with C2-Cer for 24 h. C2-Cer acted as a potent inducer of cytotoxicity in HNSCC cells, with varying sensitivities. HN30 cells were more sensitive to C2-Cer than HN4 cells under similar conditions. The inhibitory effect of C2-Cer was concentration-dependent. Compared with control cells, treated cells showed the typical characteristics of apoptotic cell death, including cell shrinkage, intracellular vacuolation, and even detachment from the culture flask. Early-stage apoptosis and necrosis increased with C2-Cer treatment concentration-dependently. PARP cleavage acts as a marker of apoptosis and is associated with the repair of DNA. Cleaved PARP was significantly increased after 24 h exposure to C2-Cer, implying the induction of apoptosis. C2-Cer has been reported to induce apoptosis in leukemia cells and colon carcinoma cells via DNA laddering [19,20] and C2-Cer was shown to act as a second messenger, mediating tumor necrosis factor-α-induced DNA fragmentation in U937 promonocytic cells [29]. The results of the present study showed that C2-Cer treatment of HNSCC cells caused DNA fragmentation within 24 h, as determined by agarose gel electrophoresis. Interestingly, cells pretreated with C2-Cer underwent necrosis rather than apoptosis, as observed from the fuzzy and continuous DNA laddering patterns. Caspase-3 protein expression showed no difference between C2-Cer-pretreated and normal cells, and viability of C2-Cer-treated cells was unaffected by the treatment with pan-caspase inhibitor. We therefore speculated that C2-Cer induced programmed cell death via a caspase-3-independent pathway in HNSCC cells. Necrotic cell death, which has traditionally been viewed as a form of passive cell death, may be executed through a mechanism termed necroptosis or programmed necrosis [30,31]. Necroptosis is a recently-identified process distinct from apoptosis and necrosis, which is specifically regulated by the death-domain-containing kinase, receptor-interacting protein 1 (RIP1) kinase. Growing experimental evidence indicates that cell death induced by the activation of the death receptor may be executed through alternative cell-death pathways, including apoptosis and necroptosis. The highly specific inhibitor of necroptosis, Nec-1, is a potent inhibitor of RIP1 kinase activity [30,32]. The current results showed that Nec1 had a positive effect on HNSCC cells viability, indicating that C2-Cer exerted its anti-tumor effect on HNSCC cells by inducing intracellular apoptosis and necroptosis via a caspase-3-independent pathway.

Autophagy occurs extensively in various stages of tumor progression. However, increasing evidence suggests that ceramides are involved in mediating two opposing autophagic pathways, which favor either cell survival or cell death [33]. Tamoxifen, which is known to inhibit glucosylceramide (GlcCer) synthase, elevates endogenous ceramide levels and induces autophagic cell death in MCF-7 cells, but also triggers protective autophagy to delay cell death in other cancer cell types [34–36]. Our results indicate that C2-Cer was able to induce autophagy in HNSCC cells, as well as apoptosis and necroptosis. We found numerous LC3-II granules scattered in the cytoplasm, especially perinuclear, after exposure to C2-Cer (50 μM) for 24 h, as well as a marked increase in the number of autophagic vacuoles observed via electron microscopy. In addition, LC3B protein levels were significantly increased by C2-Cer treatment, indicating activation of LC3B to C2-Cer exposure. We evaluated the pro-survival or pro-death effect of autophagy induction using the late-phase autophagy inhibitor CQ, which prevents autophagosomal degradation [37]. The proliferation-inhibition rate of cells treated with C2-Cer and CQ was greater than that of cells treated with C2-Cer only, suggesting that CQ could sensitize HNSCC cells to C2-Cer exposure, and that C2-Cer-induced autophagy might protect HNSCC cells from death, as indicated by other studies [34,38,39]. Although C2-Cer has been the subject of extensive research, the relevant mechanism of autophagy remains unclear. Guenther *et al*. reported that ceramides suppressed nutrient transporter protein expression, induced cell starvation, and led to AMPK-dependent autophagy induction [38]. Scarlatti *et al*. showed that exogenous C2-Cer treatment enhanced Beclin1 expression and autophagy induction, which was blocked by the ceramide synthase inhibitor myriocin, indicating that ceramide-regulated Beclin1 expression at the transcriptional or post-transcriptional level occurred [40]. We investigated the mechanism of C2-Cer-induced autophagy by determining the effects of C2-Cer on p-ERK1/2, p-Akt, Beclin1, and p-AMPK expression, and found that although p-ERK1/2 expression levels were positively related to the concentration of C2-Cer, other factors remained unaffected. Interestingly, similar to it role in autophagy, ERK1/2 has been shown to play a dual role in cell proliferation [41–45]. We determined the role of p-ERK1/2 in autophagy by treating cells with the MEK inhibitor PD98059. MEK activates mitogen-activated protein kinase to inhibit transient ERK1/2 phosphorylation. P-ERK1/2 and cell viability were significantly reduced by PD98059. P-mTOR, an autophagy inhibitor and downstream target protein of ERK1/2, showed high expression, while LC3B showed low expression, indicating that C2-Cer-induced autophagy was significantly inhibited. These results suggested that C2-Cer could induce intracellular protective autophagy via an ERK1/2 pathway. Taniguchi *et al*. reported that C2-Cer facilitated dephosphorylation of mTOR in CHO cells, and showed the involvement of a ceramide-dependent cellular signal in cell death concomitant with autophagy [46]. However, Shigeru *et al*. found that C2-Cer could induce autophagic cell death in malignant glioma cells via activation of BCL2/adenovirus E1B 19 kDa protein-interacting protein 3 (BNIP3) [47]. Exogenously-added C2-Cer might thus induce autophagy leading to distinctly different cell fates through diverse pathways. We found that C2-Cer might have a positive effect on the proliferation of HNSCC cells.

The anti-tumor effect of chemotherapeutic drugs is to compromise tumor cell homeostasis. However, the possibility of an endogenous or induced pro-survival effect also exists, and the balance between these effects determines the susceptibility of tumor cells to chemotherapy. Ceramides are vital biomolecules that are able to induce programmed cell death, and themotherapeutic agents may increase endogenous ceramide levels. We found that C2-Cer might induce a pro-survival effect through an ERK-related pathway in HNSCC. Previous studies reported that attenuation of C(18)-ceramide in HNSCC tumors correlated with lymphovascular invasion and nodal metastasis [23]. However, ERK was readily detected in HNSCC cells compared with Human Keratinocyte HaCat cells [48]. Endogenous ceramides might not be positively associated with the expression of ERK. These results suggest that ceramide may not interfere with HNSCC cells proliferation by direct regulation of the ERK pathway, but that the process may involve a negative-feedback effect of HNSCC cells under ceramide stress.

## 3. Experimental Section

### 3.1. Chemicals and Cell Culture

C2-Cer (*N*-acetyl-d-sphingosine), PD98059 (Sigma-Aldrich, St. Louis, MO, USA), and Nec-1 (Merck KGaA, Darmstadt, Germany) were dissolved in sterile DMSO and stored at −20 ºC before use (final concentration of DMSO was less than 0.1%). CQ (J & K Chemical, Beijing, China) was dissolved in double distilled water and stored at −20 ºC before use. Z-VAD-FMK (20 mM; Beyotime, Jiangsu, China) was stored at −20 ºC before use.

HN4 and HN30 human HNSCC cells (HN4, base of tongue, T4N1M0; HN30, pharynx, T3N0M0) were kind gifts from Dr. W.T. Pan, Wuhan University, China. The cells were maintained as monolayers in DMEM medium (Invitrogen, Carlsbad, CA, USA) containing 10% heat-inactivated fetal bovine serum (Gibco/Invitrogen, Carlsbad, CA, USA) and 1% penicillin/streptomycin (Gibco-Invitrogen, Carlsbad, CA, USA) at 37ºC under a humidified atmosphere with 5% CO_2_.

### 3.2. CCK-8 Assay

The proliferation-inhibition effect of C2-Cer on HNSCC cells was analyzed via the CCK-8 assay according to the manufacturer’s instructions. Briefly, cells at a density of 1 × 10^4^ cells/well were plated into a 96-well plate (100 μL/well). Cells were treated with C2-Cer (0, 20, 40, 60 μM, respectively), C2-Cer plus autophagy inhibitor CQ (5 μM), necroptosis inhibitor Nec-1 (5 μM) or MEK inhibitor PD98059 (10 μM) for 24 h, respectively, 24 h after seeding. At least five wells were used for each group. After the incubation, 10 μL of the CCK-8 reagent from a Cell Counting Kit-8 (Dojindo Laboratories, Kumamoto, Japan) was added to each well and cells were subsequently kept in culture. The absorbance was measured at 450 nm using a BioTek ELX800 microplate reader (BioTek, Vermont, NE, USA) at 1, 2, 3, and 4 h after addition of the CCK-8 reagent. The inhibition rate of cell proliferation was calculated as follows:

(1) Inhibition rate (%)=[(Mean OD of control group-Mean OD of individual test group)/Mean OD of control group]×100%

OD: optical density [49]. All experiments were repeated three times.

### 3.3. Assay for DNA Fragmentation

Cells (2 × 10^6^) were divided into two groups: a DMSO-treated group and a C2-Cer-treated group (50 μM). The cells were treated for 24 h and then collected. Fragmented DNA was extracted using DNAiso Reagent, according to the manufacturer’s instructions (Takara, Otsu, Shiga, Japan) and subjected to electrophoresis on 2 g/L agarose gels containing 0.5 g/L ethidium bromide, and visualized by UV transillumination. All experiments were repeated three times.

### 3.4. Flow Cytometry Analysis

The apoptotic effect of C2-Cer on the HNSCC cells was determined via BD LSRII flow cytometry (BD Bioscience, Bedford, MA, USA). Cells (2 × 10^5^) were incubated as described above and suqsequently collected. Cell suspensions were treated according to the experimental protocols (Alexa Fluor 488 annexin V/Dead Cell Apoptosis Kit with AlexaFluor 488 annexin V and PI for Flow Cytometry, Invitrogen, Carlsbad, CA, USA) and observed under a Leica MM AF upright fluorescence microscope (Leica, Heidelberg, Germany). All experiments were repeated three times.

### 3.5. Immunofluorescence

Immunofluorescence analysis of LC3 was performed as described previously [50]. Cells on the chamber slides were washed with phosphate-buffered saline (PBS), fixed and permeabilized with 2% acetone at 4 ºC for 15 min, and blocked with PBS containing 0.5% bovine serum albumin (BSA) for 1 h at room temperature. Cells were then incubated with anti-LC3 (1:200 diluted in BSA buffer, Sigma Chemical Co, St. Louis, MO, USA) antibody at 4 ºC overnight, washed with BSA buffer and PBS, and incubated with Alexa Fluor 488-conjugated anti-rabbit antibody (1:1000 diluted in BSA buffer) for 1 h at room temperature. The cells were then incubated with 4′, 6-diamidino-2-phenylindole (DAPI) at 4 ºC for 15 min and washed with PBS. Slides were mounted and examined using a fluorescence microscope.

Immunofluorescence analysis of necroptosis was performed using a Hoechst 33342/PI Detection Kit (Beyotime, Nanjing, China). Cells were stained in 6-well plates using Hoechst 33342 and PI successively, and images were acquired using a fluorescence microscope. All experiments were repeated three times.

### 3.6. Electron Microscopy

Samples were fixed with 2.5% glutaraldehyde solution, buffered at pH 7.4 with 0.1 M Millonig’s phosphate at 4 ºC for 2 h, postfixed in 1% osmium tetroxide solution at 4 ºC for 1 h, dehydrated in graded concentrations of ethanol, and embedded in epoxy resin (Quetol 812; Nissin EM Co. Ltd., Tokyo, Japan). Ultrathin sections (80 nm) were cut on a Reichert Ultracut E ultramicrotome (Reichert, Nu loch, Germany), stained with uranyl acetate and lead citrate, and examined with an H-7650 electron microscope (Hitachi High-Technologies, Tokyo, Japan) at 80 kV.

### 3.7. Western Blot Analysis

The cells were washed with PBS, harvested in lysis buffer with phenylmethanesulfonyl fluoride and treated by sonication. Samples containing equal amounts of protein were resolved on 8%–15% sodium dodecyl sulfate–polyacrylamide gels, transferred to polyvinylidenedifluoride membranes, and probed sequentially with antibodies against LC3B, Beclin 1, cleaved PARP, cleaved caspase-3, p-ERK1/2, p-AMPK, p-Akt, p-mTOR (pan, 1:1000; Cell Signaling Technology, Beverly, MA, USA). After incubation with the secondary antibody, the blots were developed using an enhanced chemiluminescence kit (Bioind, Kibbutz, Israel) [51]. All experiments were repeated three times.

### 3.8. Statistical Analysis

Experiments were performed at least in triplicate and the results are expressed as means ± corresponding standard deviation. The significance of differences between two variables was analyzed using Student’s *t*-tests. Statistical analyses were performed using SPSS software (ver. 16.0 for Windows; SPSS, Chicago, IL, USA). A *p*-value <0.05 was considered to be statistically significant.

## 4. Conclusions

Ceramides have recently been widely reported to exert anti-tumor effects and to show intracellular accumulation after drug treatment via *de novo* synthesis. These observations suggest their potential application in tumor therapy. In this study, we showed that exogenous C2-Cer caused caspase-3-independent apoptosis and programmed necrosis in HNSCC cells, as well as protective autophagy. These results add to a better understanding of the mechanisms of anti-tumor drug action, and suggest potential ways of enhancing chemosensitivity, which may be reduced by protective mechanisms, such as autophagy, or by special properties, such as apoptosis resistance. Short-chained ceramides encapsulated by nanocarriers and delivered intravenously or intraperitoneally have proven successful in inducing cell death without systemic side effects in multiple syngeneic and xenograft cancer models [52–55]. Ceramides may therefore represent a valuable target for further research, and a potential anticancer drug for the treatment of patients with HNSCC.
